# Lung protective characteristics of volatile anesthetic sedation

**DOI:** 10.1186/s13054-026-05998-8

**Published:** 2026-04-17

**Authors:** Maria Alejandra Alape Moya, Lloyd Chen, Nancy Otaluka, Alper Gulluoglu, Daniel Talmor, Brian O’Gara

**Affiliations:** 1 Department of Anesthesia, Critical Care and Pain Medicine, Beth Israel Deaconess Medical Center, Harvard Medical School, 330 Brookline Ave, Boston, 02215 MA USA; 2 Center for Anesthesia Research Excellence (CARE), Beth Israel Deaconess Medical Center, Boston, MA USA

**Keywords:** Volatile anesthetics, Volatile sedation, Lung protective sedation, Mechanical ventilation, Intensive care

## Abstract

This narrative review serves as an update to previous reviews on the topic after the results of recent randomized controlled trials and meta-analyses. It describes some of the latest evidence around the use of volatile sedation as an alternative to intravenous sedation in mechanically ventilated patients in the intensive care unit (ICU) and their possible lung-protective properties. Preclinical evidence supporting an anti-inflammatory protective effect of volatile anesthetics suggests that volatile sedation could be employed in patients with inflammatory lung injury, including acute respiratory distress syndrome (ARDS). Large randomized controlled non-inferiority trials of isoflurane for general ICU sedation have suggested that it is effective, with no differences between groups in safety outcomes; however, the results of a recent RCT in patients with ARDS have demonstrated significant harm with the use of sevoflurane sedation. Therefore, the utility and safety of volatile sedation in patients with inflammatory lung injury, including ARDS, is now in question.

## Introduction

The use of volatile anesthesia was first introduced into surgical practice in the nineteenth century, when Crawford Williamson Long administered ether during the excision of a neck tumor [1]. The subsequent development of fluorinated anesthetics, including isoflurane, sevoflurane, and desflurane, characterized by greater chemical stability and improved safety, established volatile anesthesia as the most widely used method of anesthetic delivery in the operating room. Volatile agents have demonstrated reliable efficacy, rapid emergence, and outcomes comparable to those achieved with total intravenous anesthesia [2]. 

In the ICU, achieving effective sedation while preserving lung-protective strategies remains a major challenge, particularly in patients requiring prolonged mechanical ventilation. Properties such as low solubility, which allows easier titration and rapid wake-up, along with minimal hepatic metabolism and renal excretion, make volatile anesthetics particularly suitable for critically ill patients who may have limited options for intravenous sedation during mechanical ventilation [3]. 

The use of volatile anesthetics in the ICU was historically limited by incompatibility with standard mechanical ventilators. This changed in recent years with the introduction of specialized delivery devices such as the Anaesthetic Conserving Device (ACD, Sedana Medical, Daneryd, Sweden) and MIRUS (TIM GmbH, Koblenz, Germany). Unlike modern anesthesia machines, which integrate a ventilator with anesthetic vaporizers, ICU ventilators are not equipped with this function. The ACD and Mirus connect to the ventilator circuit, vaporize liquid anesthetic, and recapture approximately 90% of the exhaled agent using an activated carbon reflector, enabling efficient and contained anesthetic delivery in the ICU environment [4, 5]. 

Several clinical trials have explored the potential impact of volatile sedation on ICU practice. The aim of this review is to examine the evidence surrounding lung protection from volatile anesthetics and their use as sedatives for mechanically ventilated patients in the ICU.

## Proposed mechanisms of lung protection

### Anti-inflammatory effect

#### Preclinical evidence

**Fig. 1:**
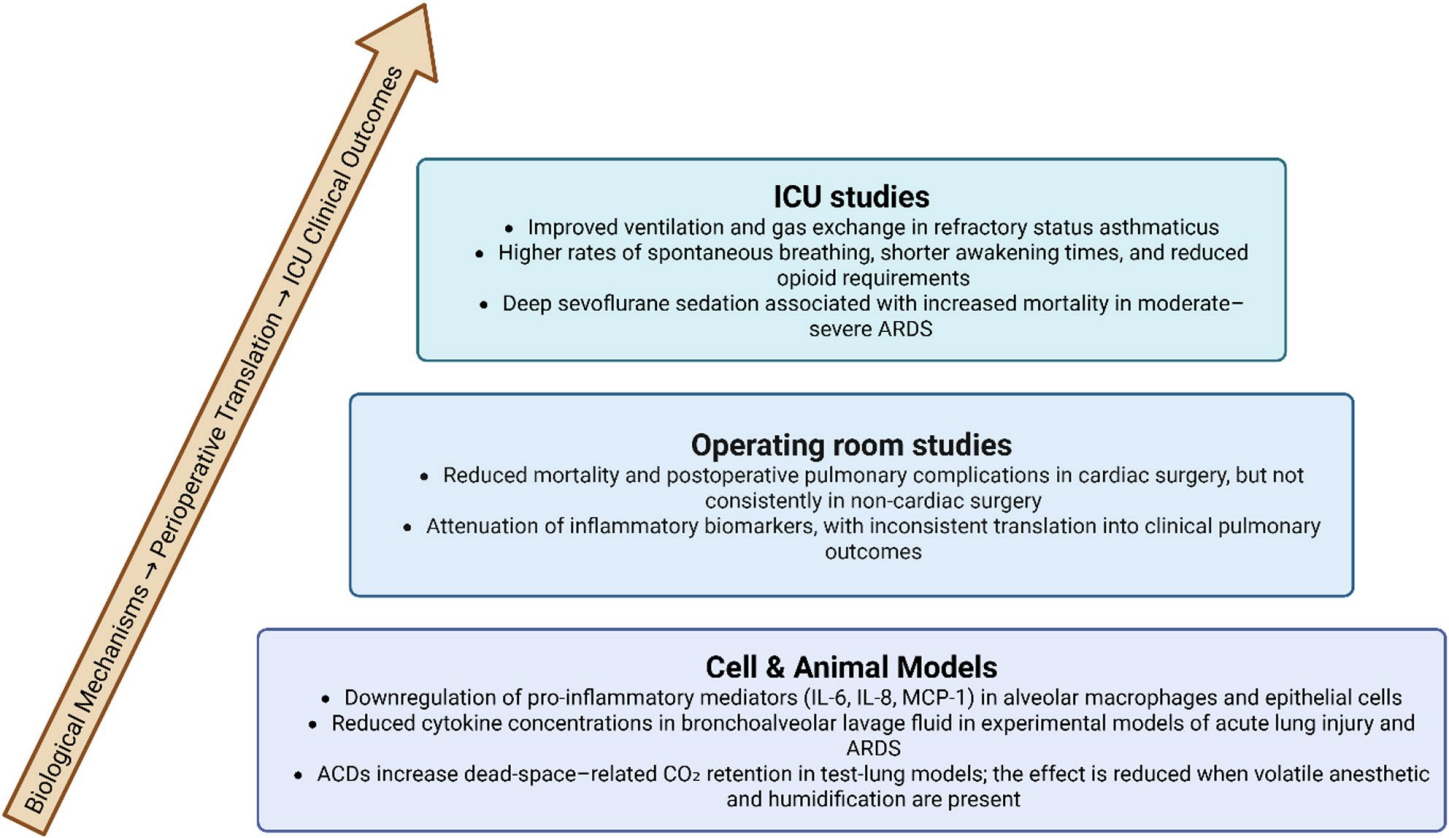
Summary of evidence regarding lung protection from volatile anesthetics in laboratory and human studies. ACD, Anaesthetic conserving device, IL, Interleukin, MCP, Monocyte Chemoattractant Protein-1

In vitro and animal models have suggested that volatile sedatives may exert anti-inflammatory effects, potentially reducing inflammatory lung injury [6], which could be beneficial in conditions such as acute respiratory distress syndrome (ARDS) (Fig. 1). In vivo studies have shown that volatile anesthetics act on different molecular targets depending on the phase of the immune response. During the recognition phase, primarily mediated by alveolar macrophages, sevoflurane downregulates the expression of pattern recognition receptors (PRRs) and NF-κB, a transcription factor involved in signaling pathways associated with ischemia/reperfusion injury. This attenuation has been linked to reduced tissue damage, although its clinical relevance has been more pronounced in renal injury during transplantation. In the recruitment phase, sevoflurane and isoflurane have demonstrated reduced chemotaxis in endotoxin-injured alveolar epithelial cells [7]. Desflurane modulates the expression of genes and proteins responsible for adhesion molecules and TNF-α, resulting in decreased recruitment of alveolar granulocytes and macrophages [8, 9]. Other mechanisms may involve regulation of inducible nitric oxide synthase (iNOS) activity and protection of the endothelial glycocalyx and tight junctions, reducing pulmonary vascular hyperpermeability [10]. 

In contrast, some studies indicate that volatile anesthetics may also contribute to oxidative stress. In healthy animal models, exposure to desflurane resulted in a significant increase in malondialdehyde (MDA) excretion, a marker of lipid peroxidation, compared to propofol, while sevoflurane showed no difference. Regarding antioxidant enzymes, glutathione peroxidase (GPX) concentrations decreased with desflurane exposure but increased with propofol, with no significant change observed with sevoflurane. Superoxide dismutase (SOD) concentrations were not significantly affected by the type of sedative [11]. These findings suggest that oxidative stress profiles vary by agent.

Disease-specific experimental models provide additional insight. In a rat study comparing sevoflurane and isoflurane in pulmonary versus extrapulmonary ARDS, the origin of lung injury appeared to influence the effectiveness of their anti-inflammatory properties, with significant effects observed primarily in pulmonary ARDS. Sevoflurane-specific effects included increased lung compliance and reduced alveolar collapse compared with baseline, suggesting a potential lung-protective mechanism through attenuation of atelectrauma [12]. This underscores the importance of the origin of lung injury, anesthetic agent, and comorbidities when evaluating the potential clinical benefit.

#### Translational studies

Although preclinical studies suggest anti‑inflammatory and lung‑protective properties of volatile anesthetics, translational models in humans and clinical trials have produced mixed results. A meta-analysis of eight randomized controlled trials including 365 subjects demonstrated a significant reduction in alveolar inflammatory markers such as TNF-α (SMD − 1.51; 95% CI −2.15 to −0.87; *p* < 0.001), IL-6 (SMD − 0.70; 95% CI −0.99 to −0.41; *p* < 0.001) and IL-8 (SMD − 1.32; 95% CI −2.20 to −0.45; *p* = 0.003) in patients receiving volatile anesthesia compared to propofol during one-lung ventilation for thoracic surgery. However, these biomarker changes did not correspond to significantly fewer pulmonary complications, although hospital stay was shorter in the volatile group (weighted mean difference [WMD] − 3.59 days; 95% CI − 5.70 to − 1.48 days; *p* = 0.001) [6, 13]. 

More recently, a randomized controlled trial evaluating the effect of sevoflurane and its lung-protective properties in cardiac surgery compared to propofol found that there was no difference in the change in TNF-α concentrations in bronchoalveolar lavage (BAL) after cardiopulmonary bypass (median [IQR] change, 17.24 [1.11–536.77.11.77] v 101.51 [1.47–402.84.47.84] pg/mL, *p* = 0.31). Yet, sevoflurane was associated with lower plasma levels of interleukin-8 (median [IQR], 53.92 [34.5–55.91] v 66.92 [53.03–94.44] pg/mL, *p* = 0.04) as well as a diminished increase in the post-bypass receptor for advanced glycation end products (RAGE) (median [IQR], 174.59 [73.59–446.06] v 548.22 [193.15–852.39] pg/mL, *p* = 0.03) compare to propofol group, proposing a reduce lung injury. However, none of the other biomarkers in blood or plasma differed between groups [14]. Regarding secondary outcomes such as postoperative pulmonary complications, there was also no statistically significant difference between groups.

In a specific ARDS population, Jabaudon et al. conducted a randomized controlled pilot study comparing sevoflurane with midazolam and found significantly lower levels of both plasma and alveolar RAGE on day 2 in the sevoflurane group. Plasma RAGE concentrations were lower in patients receiving sevoflurane (median [IQR], 1164.5 [730.7–1830.4] vs. 1968.6 [1191.6–2989.7]; *p* = 0.02), as were alveolar RAGE levels (median [IQR], 2075.6 [1216.4–7635.5] vs. 12,202.9 [3769.0–32,648.6]; *p* = 0.04) [15]. Additionally, the investigators found a significantly higher PaO_2_/FiO_2_ ratio in the sevoflurane group beginning on day 2.

It is important to recognize that preclinical studies are conducted under tightly controlled conditions, with shorter exposure times, lower anesthetic doses, and less heterogeneous injury patterns than those encountered in perioperative and ICU settings. These differences may partially explain the variability seen in clinical trial outcomes. Overall, while volatile anesthetics demonstrate anti-inflammatory properties in experimental models and some clinical contexts (Table 1), their impact on clinically relevant pulmonary outcomes remains uncertain, underscoring the need for well‑powered studies specifically designed to evaluate lung‑protective endpoints in ICU populations, particularly in ICU settings where prolonged volatile sedation could yield different results.

**Table 1:** Characteristics of translational studies that compared inflammatory biomarkers in volatile anesthetics vs. intravenous sedation

Study	Patient population (Type of study)	Volatile anesthetic used	Effect of volatile anesthetics on biomarkers	Clinical relevance
Sun and colleagues (2015)	8 RCTs, *n* = 365 patients undergoing one- lung ventilation for thoracic surgery (Meta-analysis)	Isoflurane/sevoflurane/desflurane vs. Propofol	**Alveolar**: alveolar TNFα (SMD − 1.51; 95% CI − 2.15 to − 0.87; *p* < 0.001), IL-6 (SMD − 0.70; 95% CI − 0.99 to − 0.41; *p* < 0.001) and IL8 (SMD − 1.32; 95% CI − 2.20 to − 0.45; *p* = 0.003) were lower in volatile group than those in propofol group. No significant difference for IL-1β	Volatile anesthetics exert anti-inflammatory protective effects in patients undergoing OLV for thoracic surgery
Jabaudon and colleagues (2017)	50 adults with moderate-to-severe ARDS (RCT)	Sevoflurane vs. midazolam	**Alveolar**: Significant lower day 2 sRAGE (median [IQR] 2075.6 [1216.4–7635.5.4.5] vs. 12202.9 [3769.0–32648.6.0.6] pg/mL-1, *p* = 0.04), IL-6 (median [IQR] 39.8 [35.6–485.9.6.9] vs. 474.0 [166.3–1892.4.3.4] pg/mL-1, *p* = 0.03), IL-8 (median [IQR] 50.5 [10.4–103.1.4.1] vs. 121.2 [35.3–499.6.3.6] pg/mL-1, *p* = 0.04) and TNFα (median [IQR] 58.7 [7.8–124.6.8.6] vs. 124.6 [99.9–285.8.9.8] pg/mL-1, *p* = 0.03) in the sevoflurane group. No significant difference for IL-1β **Plasma**: Significant lower day 2 sRAGE (median [IQR] 1164.5 [730.7–1830.4.7.4] vs. 1968.6 [1191.6–2989.7.6.7] pg/mL-1, *p* = 0.02), IL-6 (median [IQR]53.9 [34.9–138.9.9.9] vs. 230.4 [62.9–385.7.9.7] pg/mL-1, *p* = 0.02) TNFα (median [IQR], 1.7 [1.2–4.1] vs. 4.5 [1.8–7.6] pg/mL-1, *p* = 0.04) in the sevoflurane group. No significant difference for IL-1β or IL-8	The use of inhaled sevoflurane improved oxygenation(PaO2/FiO2 ratio mean ± SD,205 ± 56 vs.166 ± 59;p = 0.04) and decreased levels of a marker of epithelial injury and of some inflammatory markers, compared with midazolam
O’Gara and colleagues (2022)	40 adults undergoing cardiac surgery with cardiopulmonary bypass (RCT)	Sevoflurane vs. propofol	**Alveolar**: no significant difference for post-bypass TNFα, IL-1β, IL-6, IL-8, MCP-1, sICAM-1, RAGE, SP-D and BAL protein **Plasma**: Significantly lower concentration of post-bypass IL-8 (median [IQR], 53.92 [34.5–55.91.5.91] vs. 66.92 [53.03–94.44] pg/mL, *p* = 0.04) and lower median increase in RAGE for patients in the sevoflurane group (median [IQR], 174.59 [73.59- 446.06] vs. 548.22 [193.15–852.39.15.39] pg/mL, *p* = 0.03).	Sevoflurane did not consistently prevent lung inflammation or postoperative pulmonary complications compared to propofol.

RCT, Randomized controlled trial, TNFα, tumor necrosis factor alpha, BAL, bronchoalveolar lavage, IL, interleukin, MCP-1, monocyte chemoattractant protein, sICAM-1, soluble intercellular adhesion molecule, RAGE, receptor for advanced glycosylation end products, sRAGE, soluble Receptor for Advanced Glycation End products, SP-D, surfactant protein, Ang-1, Ang-2, Angiopoietin, OLV, one-lung ventilation

### Effect on the respiratory system

#### Spontaneous respiration

Maintaining spontaneous breathing in critically ill, mechanically ventilated patients is desirable as soon as clinically feasible to prevent diaphragmatic atrophy and dysfunction, which can result in a delay in weaning and extubation due to impaired respiratory muscle weakness [16]. The SEDACONDA trial was a phase 3, randomized, controlled, open-label, non-inferiority study that evaluated the efficacy and safety of up to 54 h of isoflurane compared with propofol in invasively ventilated adults in ICUs in Germany and Slovenia. A post-hoc analysis including 66 patients demonstrated that those who received isoflurane spent a significantly greater proportion of time spontaneously breathing compared with the propofol group (82% [95% CI: 69–90] versus 35% [95% CI: 22–52]; median difference: 61% [95% CI: 14–89]; *p* < 0.001). This effect persisted after adjustment for sufentanil dose and arterial carbon dioxide partial pressure (adjusted risk ratio: 2.2 [95% CI: 1.4, 3.3], *p* < 0.001) [17]. It is important to note that no patient in this study had severe ARDS, which limits the generalizability of these findings to patients with more severe forms of lung injury.

An inadequate level of sedation can result in patient-ventilator dyssynchrony, increasing the risk of lung injury [18]. Similarly, prolonged use of intravenous sedation can lead to tachyphylaxis, requiring escalating doses to achieve the desired level of sedation and often necessitating neuromuscular blockade to prevent further lung damage. Volatile sedation may provide an alternative to reduce intravenous sedative requirements while maintaining adequate respiratory effort in patients who are difficult to sedate [19]. Furthermore, volatile anesthetics could serve as a valuable option during shortages of intravenous sedatives, as experienced during the COVID-19 pandemic [20]. 

A retrospective observational study of patients with ARDS receiving sevoflurane and prone positioning reported successful transition to pressure-supported spontaneous ventilatory mode. However, limitations included patients with very low tidal volumes who did not achieve the desired sevoflurane concentration and those with cumulative exposure to opioids or other sedatives prior to enrollment [21]. 

#### Bronchodilation

Volatile anesthetics are well-recognized bronchodilators and have historically been used as rescue therapy in refractory status asthmaticus. In severe asthma, these agents are attractive because they provide deep sedation and bronchodilation, and clinical reports describe rapid improvements in hypercapnia and ventilatory mechanics consistent with reduced airflow obstruction [22, 23]. 

At the level of airway smooth muscle, volatile anesthetics such as sevoflurane and isoflurane reduce contractile tone by altering intracellular calcium signaling, resulting in smooth muscle relaxation and decreased airway resistance [24, 25]. In addition, animal and experimental models suggest that volatile anesthetics may attenuate reflex bronchoconstriction by blunting airway sensory–vagal reflexes [26, 27]. In mechanically ventilated patients with asthma, these effects can translate into lower resistive pressures at a given inspiratory flow, improved expiratory flow, and partial relief of dynamic hyperinflation and auto-PEEP. Importantly, these bronchodilatory effects occur independently of β-adrenergic pathways, explaining their utility in refractory bronchospasm when β-agonists are ineffective [28, 29]. 

Consistent with a rapid onset of action, human physiology studies have demonstrated a significant reduction in respiratory system resistance within minutes of volatile anesthetic exposure [28]. Beyond their effects on airway tone, animal and clinical studies suggest that sevoflurane may also exert anti-inflammatory effects in the airways, including attenuation of allergic airway inflammation [30, 31]. 

However, the clinical evidence supporting volatile anesthetic use in status asthmaticus is based almost entirely on very low‑certainty data from small, uncontrolled case series and retrospective reports, and no randomized trials exist. A systematic review focused on adult status asthmaticus identified 13 publications comprising 18 mechanically ventilated patients with refractory bronchospasm despite maximal conventional therapy. In most cases, initiation of sevoflurane was associated with rapid improvement in ventilatory physiology, including reductions in PaCO₂ and airway pressures with correction of respiratory acidosis, consistent with decreased airway resistance; hypotension was the most frequently reported adverse effect [32]. 

**Fig. 2:**
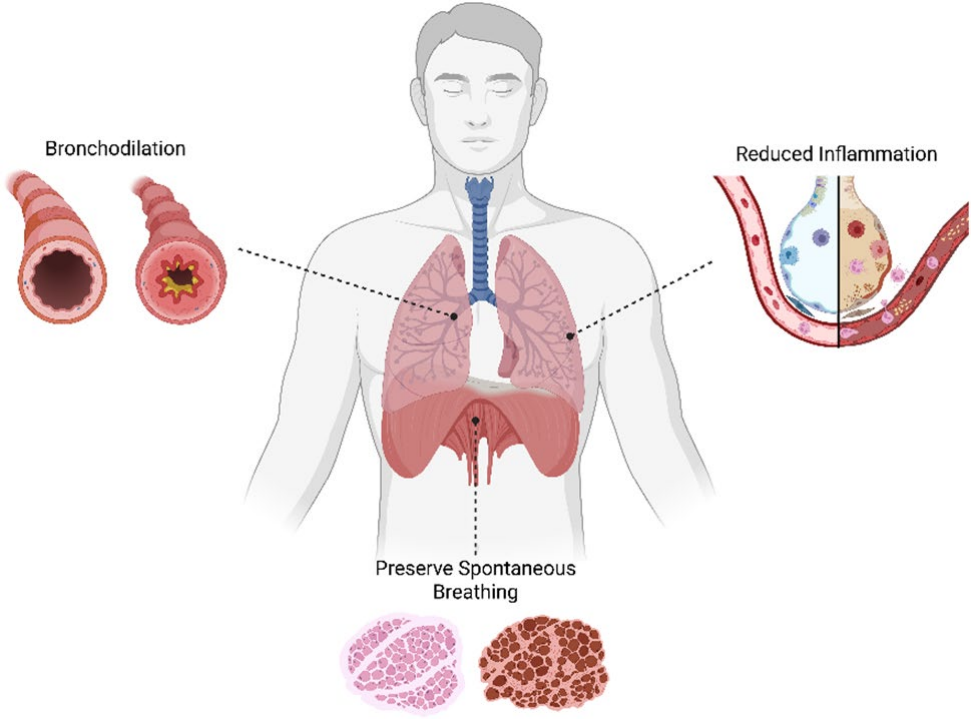
Potential lung protective effects of volatile anesthetics on the respiratory system

Status asthmaticus cases were also identified within a systematic review evaluating volatile anesthetic use in adult and pediatric ICU populations, in which volatile anesthetics were used as rescue therapy after failure of standard management and were consistently associated with improved ventilation and gas exchange, although interpretation was limited by small sample sizes, heterogeneity, and noncomparative study designs [33]. More recently, a retrospective observational cohort of 62 adults with near-fatal asthma, including patients requiring ECMO, reported volatile anesthetic use in 38 patients (61%). Despite greater baseline disease severity, patients receiving volatile anesthetics demonstrated improved ventilatory mechanics, and overall mortality was low (Fig. 2). The authors concluded that volatile anesthetic therapy is feasible and appears safe, while efficacy could not be established due to the nonrandomized design [34]. 

Together, these observations highlight short‑term physiological improvements with volatile anesthetics in case series of patients with refractory asthma, but uncontrolled case series are not sufficient to make efficacy claims, and without evidence from clinical trials the certainty of the available evidence remains very low.

#### Ventilatory efficiency and mechanical load

Anesthetic reflecting devices used for volatile sedation in the ICU have been shown to increase CO_2_ levels in bench studies. Although the primary driver of this increase is internal volume–related dead space ventilation, CO_2_ reflection is also a contributing factor. Two bench studies using test lungs demonstrated a significant increase in CO_2_ with the use of volatile sedation devices [35, 36]. However, the CO_2_-reflecting properties of anesthetic reflecting devices are diminished in the presence of volatile anesthetics and under humidified conditions [37]. 

Clinical interventions to correct elevated CO_2_, such as increasing respiratory rate and/or tidal volume, may lead to higher mechanical power and energy exposure to the lungs [36]. Whether these changes are clinically meaningful or sufficient to contribute to VILI or adverse outcomes remains uncertain.

### Clinical trial data

The hypothesis that different anesthetic agents may exert protective effects against postoperative pulmonary complications, supported by multiple experimental studies, prompted a meta-analysis evaluating their impact in the operating room. Although conducted in the perioperative setting, the findings were later considered relevant to ICU practice, given a similar mechanism of ventilator‑associated lung stress and the frequent postoperative transition of these patients to the ICU. This analysis included 7,104 patients from 68 RCTs undergoing general anesthesia for cardiac and non-cardiac surgery, comparing volatile anesthetics with total intravenous anesthesia (TIVA). In cardiac surgery patients, the use of volatile anesthetics was associated with a lower incidence of postoperative pulmonary complications compared with TIVA (OR = 0.71; 95% CI, 0.52–0.98; z = 2.07; *p* = 0.038; I² = 6.0%), whereas no statistically significant difference was observed in non-cardiac surgery patients [38]. Although heterogeneity was considerable and the cardiac surgery subgroup was relatively small, resulting in limited statistical power, these findings served as hypothesis-generating data for further investigation of the potential organ-protective effects of volatile anesthetics in the intensive care unit setting.

Sedation using volatile anesthetics was first studied in the ICU population in 1989. A randomized trial comparing midazolam and isoflurane (*n* = 30 per group) up to 25 h found satisfactory sedation for a greater proportion of the time (86% vs. 63%) and earlier tracheal extubation for the isoflurane group [39]. This was validated by Sackey et al., which randomized 40 ICU patients to either isoflurane or midazolam. The isoflurane group demonstrated significantly shorter mean wake-up times (10 vs. 110 min to follow commands) and time to extubation (10 vs. 252 min) [40]. The SEDACONDA study compared isoflurane (*n* = 150) to propofol (*n* = 151) for up to 54 h and found significantly shorter median wake-up time with isoflurane (median [IQR], 20 [10–30] vs. 30 [11–120] min; *p* = 0.0011) [41]. A 2017 meta-analysis (13 trials, 1,027 patients) found that volatile sedation shortened awakening time by 80 min and extubation time by 196 min compared to IV sedation (propofol or midazolam) [42]. 

Several studies have noted a significant opioid-sparing effect, attributed to the analgesic properties of volatile agents. A retrospective study of 11 mechanically ventilated COVID-19 ARDS patients sedated with midazolam and sufentanil for a median of 82 h and then transitioned to isoflurane. Patients had significantly lower sufentanil consumption (17.3 ± 5.0 vs. 10.6 ± 4.0 µg/h; *p* = 0.005) with equivalent levels of anesthesia [43]. In another study evaluating severe ARDS patients on venovenous extracorporeal membrane oxygenation, isoflurane sedation compared to intravenous sedation significantly lowered opioid dosage: (fentanyl: 1.41 ± 0.57 vs. 1.63 ± 0.54 µg/kg/h [*p* < 0.001]; remifentanil: 0.07 ± 0.04 versus 0.14 ± 0.07 µg/kg/min [*p* = 0.005]) [44]. Consistent with these findings, the SEDACONDA trial demonstrated that patients in the isoflurane group received significantly lower total opioid equivalents than those receiving propofol with comparable pain scores, with no differences in safety endpoints, including serious adverse events, ventilator-free days, or delirium [41]. 

Based on preliminary results demonstrating that sevoflurane sedation lowered alveolar injury biomarkers and improved oxygenation in patients with ARDS, a group of investigators then conducted the SEdation with Sevoflurane in ARDS (SESAR) trial which was a multicenter, open-label RCT conducted in 37 French ICUs in which patients with moderate-to-severe ARDS (*n* = 687) were randomized to sevoflurane or propofol sedation for up to seven days [45]. The primary outcome was the number of ventilator-free days (VFDs) at 28 days. Both groups had a median of 0.0 VFDs (sevoflurane: 0.0 days [IQR, 0.0–11.9]; propofol: 0.0 days [IQR, 0.0–18.7]), indicating that most patients remained ventilated or died within the first 28 days. However, statistical analysis showed that patients in the sevoflurane group had approximately 2 fewer VFDs compared with the propofol group (median difference [95% CI] − 2.1 days [− 3.6 to − 0.7], HR [95% CI]: 0.76 [95% CI, 0.50–0.97]). Furthermore, 90-day mortality was higher in the sevoflurane group (HR [95%CI] 1.31 [1.05–1.62]). The investigators also found that patients sedated with sevoflurane had higher vasopressor requirements and a higher incidence of acute kidney injury than patients sedated with propofol. Prolonged use of sevoflurane has previously been found to increase the risk of high-output renal failure in patients with COVID ARDS [46]. 

This was an unexpected result given the findings from the same group’s preliminary study; however, this speaks to the importance of adequately powered, well-controlled trials. It is important to note some caveats to the way sedation was carried out in SESAR. Study patients underwent deep sedation with neuromuscular blockade for a median of 5 days, neither of which is currently recommended by international societies for general ICU sedation or for patients with ARDS. Based on previous knowledge of the preservation of spontaneous breathing with the use of volatile sedation, even at deeper planes of sedation, it is possible that further attempts at deepening sedation or neuromuscular blockade could have been employed during SESAR to eliminate spontaneous breathing, subjecting patients to increased risk from the hemodynamic effects of volatile anesthetics at higher doses [47, 48]. 

In relation to potential increases in mechanical power and CO₂ retention attributed to the anesthetic‑conserving device used, SESAR supplemental data suggested a potential increase in tidal volume, respiratory rate, and ventilatory ratio in the sevoflurane group, as well as higher PaCO_2_ and airway resistance. These observations were purely descriptive and were not subjected to hypothesis testing. The existing evidence remains limited, and the clinical impact of anesthetic‑conserving devices on mechanical load is still unclear.

Yamamoto et al. summarized the latest clinical evidence in a meta-analysis of randomized controlled trials, including the SESAR trial and 21 additional RCTs, with a total of 2,367 patients. Using mortality as the primary outcome, volatile anesthetics were associated with increased mortality compared to intravenous sedation at the longest follow-up (262/1107 vs. 218/1106; relative risk: 1.17; 95% confidence interval, 1.02 to 1.35; low certainty), an effect primarily driven by the SESAR trial. Regarding secondary outcomes, including ICU length of stay and duration of mechanical ventilation, the evidence was sufficient to rule out clinically meaningful reductions favoring volatile sedation, except for a modest decrease in the time from sedation cessation to extubation (mean difference, − 90.62 min; 95% confidence interval, − 124.64 to − 56.60; low certainty) [49]. The main finding of increased mortality for volatile sedation in this meta-analysis did not meet the required sample size for statistical certainty, meaning it cannot be considered definitive or generalizable to the broader ICU sedation population.

Notably, published trials have not demonstrated increased mortality with short-term exposure to isoflurane, whereas concerns have raised with longer durations of sedation using sevoflurane in ARDS, as reflected in SESAR. Although questions remain about potential differences between isoflurane and sevoflurane or the effects of shorter exposures, these distinctions are still hypothesis‑generating and highlight the need for adequately powered trials capable of evaluating agent properties and duration‑specific effects. Two recent multicenter randomized controlled trials in the United States comparing isoflurane with propofol in adults undergoing mechanical ventilation (NCT05312385 and NCT05327296) have been completed and will provide further data on the important clinical question of whether volatile sedation leads to worse mortality in the general ICU sedation population.

## Conclusions

The volatile anesthetics have been found to decrease lung inflammation and injury in preclinical models and human studies, have been used for decades to support patients with severe asthma, and to preserve spontaneous breathing in patients with respiratory failure. All of these characteristics make volatile sedation an attractive candidate for sedation of severely lung-injured patients. However, a recent large, well-controlled clinical trial found evidence of significant harm from prolonged deep sedation with sevoflurane as compared to propofol for patients with moderate to severe ARDS (Table 2). Although there are elements of the sedation protocol in SESAR trial that may have contributed to this risk, and there is a possibility that a different outcome may have been found with the use of a different volatile agent, such as isoflurane, these findings make it challenging to recommend the use of volatile sedation for the purposes of lung protection at this time until further evidence arises. Future directions should include multimodal strategies and personalized sedation protocols, supported by adequately powered randomized controlled trials to clarify the role of volatile sedation in improving outcomes for critically ill patients.

**Table 2:** A comparison of the pros and cons of volatile sedation in patients with lung injury

Pros	Cons
**Device related**	
Devices like ACD can integrate with ICU ventilators	Requires specialized devices, scavenging, and staff training, and may increase costs
Useful when intravenous sedatives are scarce (e.g., drug shortages)	Adds circuit dead space (e.g., ACD‑S ≈ 50 mL), which can increase CO_2_ retention and require an increase in tidal volume and respiratory rate, resulting in mechanical power.
**Pharmacological properties**	
Preclinical models demonstrate an anti-inflammatory effect, including in ARDS	This has not been translated fully into clinical trials
Preservation of spontaneous breathing may enable weaning	Maintaining spontaneous respiratory drive is not always beneficial, as in cases of ventilator dyssynchrony
Historical use in status asthmatics, bronchodilator effects	Studies with very low certainty (small case series). Limited clinical trial data especially in ARDS
**Clinical outcomes**	
May reduce the need for opioids, rescue sedatives, and paralytics	May need opioids to suppress respiratory drive if not desirable
In general ICU cohorts (non‑ARDS), systematic reviews suggest faster awakening/extubation with volatile sedation vs. intravenous sedatives [50]	In moderate–severe ARDS, the SESAR RCT (sevoflurane vs. propofol) showed fewer VFDs and fewer ICU‑free days
In SEDACONDA trial, isoflurane was non‑inferior to propofol for sedation; post‑hoc showed more time in spontaneous ventilation with isoflurane.	Prolonged, deep sedation with sevoflurane increased mortality in moderate to severe ARDS (SESAR trial)

ACD, anaesthetic conserving device, ARDS, Acute respiratory distress syndrome. RCT, Randomized clinical trial, VFDs, Ventilator-free days

## Data Availability

No datasets were generated or analysed during the current study.

## Abbreviations

ICU: Intensive care unit
ACD: Anaesthetic conserving device
ARDS: Acute respiratory distress syndrome
PRRs: Pattern recognition receptors
iNOS: Nitric oxide synthase
MDA: Malondialdehyde
GPX: Glutathione peroxidase
SOD: Superoxide dismutase
BAL: Bronchoalveolar lavage
RAGE: Receptor for advanced glycation end products
IQR: Interquartile range
PEEP: Positive end expiratory pressure
PaCO₂: Arterial partial pressure of carbon dioxide
TIVA: Total intravenous anesthesia
SESAR: SEdation with Sevoflurane in ARDS trial
VFDs: Ventilator-free days
